# Diverse and Complex Challenges to Migrant and Refugee Mental Health: Reflections of the M8 Alliance Expert Group on Migrant Health

**DOI:** 10.3390/ijerph17103530

**Published:** 2020-05-18

**Authors:** Danny Sheath, Antoine Flahault, Joachim Seybold, Luciano Saso

**Affiliations:** 1 Institute of Global Health, Faculty of Medicine, University of Geneva, 1202 Geneva, Switzerland; Antoine.Flahault@unige.ch; 2 Charité, Universitätsmedizin Berlin, 10117 Berlin, Germany; joachim.seybold@charite.de; 3 Faculty of Pharmacy and Medicine, Sapienza University of Rome, 00185 Rome, Italy; luciano.saso@uniroma1.it

**Keywords:** migration, mental health, refugee, global health, depression, mental illness, displacement

## Abstract

Forced migration is likely to continue to grow in the coming years due to climate change, disease outbreaks, conflict, and other factors. There are a huge number of challenges to maintaining good health, and specifically good mental health, among migrants at all stages of migration. It is vital to fully understand these diverse challenges so that we can work towards overcoming them. In 2017, as a response to the growing health challenges faced by migrants and refugees, the M8 Alliance created an expert group focussing on migrant and refugee health. The group meets annually at the Sapienza University of Rome, Italy, and this article is based on the discussions that took place at the third annual meeting (6–7 June 2019) and a special session on “Protecting the Mental Health of Refugees and Migrants,” which took place on 27 October at the World Health Summit 2019 in Berlin. Our discussions are also supported by supplementary literature to present the diverse and complex challenges to the mental health of migrants and refugees. We conclude with some lessons learned and hope for the future.

## 1. Introduction

At a time when much of the Western press seem increasingly concerned about the problem that migration poses to their way of life, what these host nations stand to gain from the entry of skilled migrants and the scale and diversity of challenges faced by migrant and particularly refugee populations is still largely underestimated. Migration is an extremely complex process with many drivers and components, each bringing a unique set of challenges, particularly in terms of health. In response to the growing health challenges faced by migrants and refugees, members of the M8 Alliance launched an annual expert meeting on migrant and refugee health [1,2]. This report is based on the discussions of the third such meeting (held at Sapienza University of Rome, Italy, 6–7 June 2019) and a special session on “Protecting the Mental Health of Refugees and Migrants,” which took place on 27 October at the World Health Summit 2019 in Berlin. The meetings draw on the 28 academic members of 19 countries that form the M8 Alliance of Academic Health Centres, Universities and National Academies in order to deliver science-based solutions to health challenges. The M8 Alliance is the academic foundation of the World Health Summit and was formed at the inaugural addition of the summit in 2009. The M8 Alliance aims to set the agenda for global health improvement and development of science-based solutions to global health challenges. For these particular meetings, the M8 alliance focussed on some of the key issues for migrant health. This report summarises their discussions, confronting the major challenges and solutions for mental health, education and access to quality mental health care services for migrants and refugees.

Reasons for migration can be numerous including social, political, economic and environmental. However, for the purposes of clarity within this report, we will discuss issues, which, whilst they may affect all migrant populations, specifically or disproportionally affect those who have been forcibly displaced. Among forced displacements in the world in 2017, we can count 25.4 million refugees and 3.1 million asylum seekers that had been forced to leave their homes and seek international protection in another country [3]. Forced migration, which refers to involuntary movement of individuals, and its implications have long been an issue for low- and middle-income countries (LMICs), with developing countries also hosting 85% of the world’s refugees [3]. However, recent migratory pressures in high-income countries (HICs) have attracted an unprecedented level of attention and concern regarding this complex matter, which has become truly global. This attention has unsurprisingly given rise to a great number of myths surrounding the so called “migrant crisis”. The UCL-Lancet Commission on Migration and Health, launched in 2018, has made an effort to dispel some of these myths about migration, including, for example, the fact that, contrary to what the headlines might have us believe, global migration as a proportion of global population is stable [4]. TEXT REMOVED Another perhaps surprising revelation is that international migrants in high-income countries (HICs) have, on average, lower mortality than the host country population [5]. What is clear is that forcibly displaced groups are still increasing in certain contexts, but it is more useful and constructive to look at migration not as a crisis or an acute event but as a disruptive movement that is changing our societies and is almost certainly here to stay.

The complexity and breadth of migrant health challenges are far too vast to cover in a single report. Hence, within this report, some of the really key issues, particularly surrounding migrant mental health, will be explored. One such issue that limits the quality of care that migrants receive is that of cultural competence, or a lack thereof. Cultural competence is defined as the incorporation of personal cultural diversity experience, awareness, and sensitivity into everyday clinical practice [6,7]. Providing culturally competent care has been associated with improved provider–client communication [8], higher satisfaction with care [9], and health status improvement. This is primarily because it promotes full comprehension of health status, adherence to medications and lifestyle recommendations, and appropriate utilisation of the health system [10]. As a patient group, migrants are particularly susceptible to suffering as a result of a lack of cultural competence from caregivers, due to their diverse cultural backgrounds. As such, improving cultural competence and global health knowledge in health worker training is likely to have particularly positive impacts on migrant health outcomes and patient satisfaction. An area of health care where cultural competence is of huge importance is in mental health, where being able to empathise with and understand your patient is key to good diagnosis and management. Mental health remains among the key challenges to migrant and refugee health [2], and is a domain that is both complex and challenging to address.

## 2. Migrant Mental Health

Migration, whatever the driver, presents a potential cascade of mental trauma—at the very least, generating anxiety and stress—and, as a result, migrants as a population group are particularly vulnerable to developing mental health disorders [11]. It is therefore essential to try to understand the complexity and range of causative factors and psychological conditions that migrants and refugees are faced with and ensure that appropriate mental health care provisions are available to help them. In order to fully understand these factors, it is important to realise that migration is not a single event but a process, which is made up of a series of spatial and temporal phases—all of which have their own features and challenges [12]. During all of these phases, psychological trauma can occur—in some cases, in a chronic fashion, leading to severe mental health conditions. From experiences of violence at displacement from their home countries and during the initial flight to mistrust towards the destination society and other refugees, these all have the potential to cause physical and psychological health problems. Whilst the mental health implications of violence within place of origin and throughout the initial fleeing have been well studied [11,13,14,15], what remains poorly understood is the psychological factors in the often lengthier processes of arrival in host countries and eventual settlement or return to home countries. Throughout these latter phases of migration, uncertainty is rife. Waiting for asylum status decisions, uncertainty related to residence status and perceived arbitrariness of the application of laws perpetuates problems of mistrust among refugees and towards members of the host society, which might lead to social isolation, conflicts and violence. Further, indecision over status means not having access to the labour market or studies, leading to inactivity and too much free time. Earlier studies have shown that meaningful activity (employment, studies or voluntary work) is important for good mental well-being, whilst boredom and inactivity can harm both mental and physical health [15,16].

Even when a decision is made concerning the status of a migrant in the country of arrival, access to the labour market and residency is often short term. Limited residence in the destination country can understandably make migrants question whether there is any point in trying to learn the local language and fully integrate if they are unsure whether they will be able to stay. This often leads people to work in jobs for which they are overqualified in order to make money to save or send home, but does not allow them to realise their full potential within these societies, meaning both the migrant and host communities miss out. At the policy level, there is a need to provide stability and a long-term perspective of stays for refugees, since currently commonplace piecemeal approaches reinforce uncertainties and do not promote good mental health [17]. Framing refugees as temporary migrants in public discourses is also counter-productive to real societal integration. Next to physical and psychological health, the social health of refugees is also important for individuals and their interactions in order to maintain the functioning of societies and social cohesion. Arrival into a different cultural context often leads to a loss of social status and these confounding factors are likely to have serious social health implications. 

Children younger than 18 years old represent 52% of total refugees worldwide [3]. In general, children constitute a vulnerable group as they rely on adults for their subsistence, protection, development and well-being. As circumstances during forced migration may compromise these basic rights, children of refugees represent an even more vulnerable group. We also know that child refugees are more at risk of presenting and developing psychological problems, although studies have shown prevalence to be quite variable [18]. The main mental health conditions encountered in children are post-traumatic stress disorder (PTSD), depression, anxiety and conduct disorders, but they remain an understudied group in this domain [19]. Establishing a reliable psychological diagnosis remains quite challenging in paediatrics, not only because of the cultural considerations but because children tend to exhibit various symptoms rather than fulfilling specific diagnostic criteria. There is also no specific screening tool currently recommended in child refugees. The best practice at this moment is the use of an invalidated screening tool and this is advised as part of refugee health assessment, but these tools usually focus on PTSD through phases of migration [20]. Because of these challenges associated with mental health diagnosis and possible inaccuracy, recent efforts have focussed rather on the identification and study of protective and risk factors for mental health among young refugees. These factors may be studied according to various models, such as the chronological model, as factors related to the three phases of migration (pre-departure, travel and settlement) contribute to the cumulative stress experienced by children and may negatively impact their mental health, with exposure to trauma in the early phase linked to worse outcomes [18].

Clinically, it is essential for health professionals caring for children of refugees, whether in temporary settlements or in high-income countries, to acknowledge the mental health risks these children incur and identify factors of vulnerability in order to respond to their specific psychological needs. Children’s exposure to traumatic events in any phase has been associated with psychological dysfunction, as has parental psychological state [18]. This highlights a need for better coordination between adult and paediatric mental health care professionals. 

Perceived health status in young refugee populations is poor and studies suggest a high prevalence of mental health problems in these groups [21]. For both health professionals and refugees, challenges such as access to a safe environment, housing, healthy nutrition, clean water, financial stability, access to work and education opportunities, which seem like physical challenges, are prioritized over challenges, which seem more psychosocially oriented. We know that refugees are at increased risk of mental health problems [19] and it has been indicated that social determinants of health, stigma, discrimination, adverse events and other challenges in the host country can be as important for mental health as traumatic events experienced during displacement or travel [17]. Hence, while physically “being safe” in the host country is important in the short term, it is not enough to promote good mental health in the long term. When planning or delivering health care for refugees, physical health problems, especially communicable diseases, always have a priority for decision makers and service providers. This is understandable in the short term, but not in the medium and long term. Today, the majority of refugees are located in low- and middle-income countries (LMICs) with limited resources for mental health care [3]. This fact, combined with (i) a high prevalence of mental problems, (ii) a tendency for migrants to rarely seek help for mental health problems, and (iii) a lack of access to quality mental health care leads to a significant and hidden mental health burden [21]. Turkey has the highest refugee population in the world, and studies here have shown, as in other countries, that despite a high prevalence of mental health problems among refugees, delivery, access to, and utilisation of mental health care services are much lower than needed [21]. A study in which quantitative data were collected via household surveys of 420 adult refugees in Ankara, Turkey, and qualitative data were collected via in-depth interviews with 10 health care providers and 10 health policy makers revealed some drivers and the scale of the mental health challenges here [21]. The study group was highly traumatized—44% had lost a family member in the war, whilst 40.0% had witnessed a murder, and the probable rates of PTSD and depression were 36.5% and 47.7%, respectively. In addition to the direct effects of war-related traumas, they were negatively affected by the living conditions in the host country and the disruption of social support systems [21]. Among the refugees who were in need of mental health care, only 9.7% were found to have used mental health services. The most common barriers experienced were language problems, cultural differences and a lack of knowledge about existing services. Almost all professionals reported that mental health services for refugees were inadequate and not easily accessible [21].

### 2.1. Migrant Mental Health and Aging

**Figure 1:**
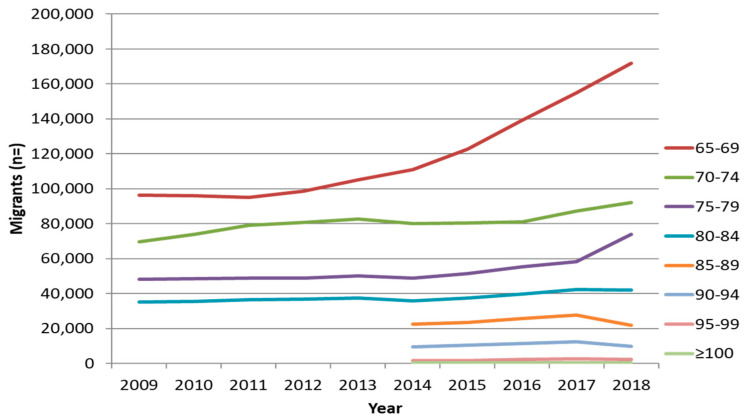
Number of migrant subjects living in Italy by age class [24] (credit to Marco Canevelli).

Globally, the human population is an aging one, so too are migrant populations aging (Figure 1). With an aging migrant population comes a whole new range of health challenges, including an increase in the dementia and cognitive disturbance case rate [4,22]. A European study of dementia centres found that diagnostic evaluation of migrant patients was challenging in 64% of the centres, mainly because of “communication problems and lack of adequate assessment tools” [23]. There is a lack of cultural competence and sensitivity in these dementia services, perhaps in part contributing to the fact that migrants are less likely to receive dementia diagnosis than natives are [23]. Immigrants also tend to present later, in part due to a lack of knowledge about available services, leading to more chronic late onset management and therefore poorer outcomes. Therapeutic treatments for some migrants diagnosed with dementia are also limited due to a lack of evidence on side effects and effectiveness of common dementia drugs on various minority groups. Return of aging populations to their native country is much understudied in Europe, which may constitute a missing group in consideration of dementia prevalence. All of these factors clearly indicate a need for far more research into the mental health of aging migrant populations, specifically with regard to dementia. 

### 2.2. Challenges to Mental Health Care for Migrants

Unfortunately, even for migrants with common mental disorders who are able to access health care facilities and seek clinical diagnoses or interventions, there is a lack of parity between the treatment that they and native residents receive [25]. Immigrants in Europe are at higher risk of forced or involuntary psychiatric interventions than are natives. The reasons for this include cultural, ethnic and language barriers, leading to a breakdown in communication between immigrant patients and mental health care professionals [25]. Whilst, in the immediate term, this is obviously highly undesirable, it also has often overlooked long-term implications due to challenges with adherence. In some settings, there is also a higher rate of physical restraints being used among migrant patients, which again is likely to reflect these barriers. Physical restraints increase the risk of harm to the patient, as stress becomes more internalised and depressive, and hence specific strategies need to be put in place to prevent this trend in the care of migrants [26]. In Italy, steps have been taken to try and improve interactions with migrants who present at psychiatric wards by employing cultural negotiators. Whilst this is a proactive and clearly much needed step, the practice is somewhat thwarted by the fact that these negotiators do not work nights or Sundays, failing those patients in need at these times. Hospitals in Italy are also investing in the further education of staff on the specific needs of migrant patients, but it is clear that these steps need to be seen as the first steps taken and more work is needed in this domain in order to ensure the mental well-being of these vulnerable groups. 

In order to fully understand the unique therapeutic challenges that mental health care workers face when dealing with refugees, it is crucially important to gather first-hand accounts from them. The following experiences were revealed by a number of therapists in Paris, France, who have pooled findings from their sessions with multiple refugees. Patients find it difficult to engage and it is therefore hard to establish a strong narrative, and this is in part due to chronic fatigue caused by a lack of sleep. This also impacts their cognitive function and capacity to learn the language of the host nation. Often patients will refuse to share details about their experiences of extreme trauma—for example, during encampment or imprisonment. Patients complain of interrupted sleep in part due to nightmares as a result of their experience. Dream sharing can provide a window into these experiences—for example, in order to highlight specific occasions when attacks or abuse has occurred. Sometimes the mind can block out these traumatic experiences, making therapy more challenging. After a session of sharing, you would normally follow with a session where you can dive into these traumatic experiences to analyse the learnings. Unfortunately, in some cases, your patient is returned to a detention centre or their home country before this can take place. These therapists have no power in this instance, which naturally leads you to question whether having a patient open up about their experiences even in these short windows helps them? Having little idea of how long you have with patients before they might be forced to leave adds another challenging dimension to the mental health care of migrants.

### 2.3. Progress in Understanding Migrant Mental Health Care Challenges

Fortunately, these challenges are increasingly being recognised and there is a huge amount of effort currently underway through a number of national and international projects to try and improve our understanding of the mental health of migrants and improve individual outcomes. These projects focus not only on mental health care for migrants but also understanding the root causes of mental health problems and training mental health care professionals to build capacity in the field. For example, Transver [27] is a Berlin-based project that provides training and education for mental health workers including volunteers. Transver has a database that lists all of their services on offer, as well as the available languages. Another German-based initiative is the Zip ethnopsychiatric ambulance service [28], a part of the general psychiatric outpatient ward for the non-German-speaking population—43% of which are refugees. Zip comprises a multi-professional team, including language and cultural interpreters, with staff from diverse cultural backgrounds. They offer single and group therapy sessions (e.g., young male Afghans, Arabic-speaking patients) with a psychodynamic approach. 

A number of successful projects do not limit themselves to dealing solely with migrants. The Horizon 2020 project, forced displacement and refugee–host community solidarity (FOCUS), is designed to significantly increase our understanding of key dynamics in refugee–host community relations and to develop and test innovative solutions for psychosocial and labour market integration. Surveys are carried out on both host and migrant populations, listening to both sides and trying to bring them together. Similarly, “Vom Schönen Leben” is a reflective citizen approach in a neighbourhood of southern Berlin with a majority of elderly Germans and a large group of comparatively younger newcomers from Syria and Afghanistan. By bringing these communities together, it is hoped that it will reduce isolation and tackle misconceptions about the respective parties. There is a call for more “one mental health” approaches, which consider the mental health of refugees with the mental health of host communities. Mental health professionals are all linked and by developing strategies that do not focus on one specific community, which leads to suspicion and segregation, you consider mental health in the population as a whole in order to prevent discrimination. Among the key challenges for migrants and refugees in Europe is exclusion or self-exclusion—this is particularly true in children, where they consider themselves as different from their native peers. The children of migrants and refugees are often suffering mental health problems for the same reasons as their parents, including traumatic experiences. Therefore, Europe needs a solution for children who are at present mostly non-visible to the systems of medical, social and educational care. Mental health interventions and education within schools can benefit all children, not just migrants—as such, it needs to have more prominence in the political agenda and deserves to be recognised by policy makers. 

## 3. Access to Mental Health Services

Access to health is a basic human right for all (United Nations Charter, 1951). As such, the final section of this report will focus on access to health care for migrants and refugees. It is important to fully understand the five dimensions of access in order to identify challenges to access (Table 1). Migrants are faced with a whole host of unique potential barriers in accessing care, including but not limited to language, culture-related differences in symptom presentation and insufficient knowledge about the structure of the health care system, as well common barriers to access including affordability, physical accessibility and quality. Despite the UN International Covenant on Economic, Social and Cultural Rights (ICESCR), guaranteeing “the right of everyone to the enjoyment of the highest attainable standard of physical and mental health,” the terrible reality is that there is a growing trend of states limiting access to health care for migrants, despite their own commitments to provide “health for all.” [4]. Migrants should be explicitly included in Universal Health Coverage (UHC) commitments to challenge the mainstream views of a health system defined by geopolitical boundaries, rather than people’s needs regardless of their official status. Creating health systems that integrate migrant populations will benefit entire communities, with better health access for all and positive gains for local populations, as insurance gaps and access challenges are not just experienced by migrants. For returning citizens/residents, there can be lengthy delays in re-accessing care provisions, and for temporary residents, such as students, they might face private insurance caps, creating treatment gaps. 

Migrants are less prevalent in France compared to the rest of EU. However, the challenges they face are the same, as are the barriers to health access. According to the World Health Organisation (WHO), undocumented migrants have the right to health care access in France, but the reality is quite different—even as recently as 2007, France had absolutely no national policy relating to migrant health and access to care [30]. Psychiatric emergencies make up approximately 40% of hospital presentations among migrants, but there are very few specialised psychiatric emergency services available in France, which already makes access challenging. Now, there is an effort by the government to remove psychiatric cover from the state medical aid for undocumented migrants, further harming their chances of accessing appropriate care. It is not only France who are failing their migrant population needs in terms of access to health care, Italian immigration policies recently changed. Since October 2018, migrant shelters have only been reserved for refugees and unaccompanied children, while asylum seekers, even if granted temporary documents, are not hosted in government shelter systems. Reassurances have repeatedly been made that new policies do not include any measure impacting migrant health care, but being hosted in shelters previously ensured that asylum seekers had access to sanitary structures, cultural mediators, and medical visits including screening procedures. At present, there are no dedicated health care workers in place to care for those unofficial arrivals, and the short- and long-term impacts of this on migrant welfare are likely to be profound. 

If access to health care is found wanting upon arrival and settling in host countries, it is no surprise that access to essential care is an even more acute challenge during the actual process of migration. Recent deaths in transit and detention centres highlight the susceptibility of immunocompromised (and malnourished) migrants. Patients with chronic disease that migrate overland (or water) have specific needs in transit and require care immediately upon resettling—one such vulnerable group are paediatric oncology patients [31]. Predictably, for undocumented migrants, it can prove extremely difficult to access the care they need even when they reach their destination, and hence their long-term prognosis is often poor. 

Health access or lack thereof can even be a driving factor of migration. Venezuela is in the midst of a mass emigration, with 2.4 million people fleeing the country between 2014 and 2018 [32]. The chief driver of this is the poor economic climate due to the rapid reduction in oil production in the country. Regular migration started happening after 2016 due to a lack of food but then the Venezuelan government closed the borders, forcing illegal migration. This financial crisis put huge strain on the health sector, to the point where Venezuelan doctors earned less than a dollar a day—the worst medical salary in the world. Professional medical associations estimate that at least 20,000 health professionals have left Venezuela in recent years. This reduction in professionals puts health services under pressure and increases the chance that the population as a whole will have limited access to adequate health care and, as in most circumstances, the most vulnerable are hardest hit [33]. This “brain drain”, as it is often termed, of skilled health workers coupled with the collapse of the country’s economy has led to a health crisis in Venezuela, characterised by a rise in infant mortality and vaccine-preventable diseases as well as the re-emergence of diseases resulting from unregulated migration [34]. People are migrating for treatment due to a lack of access to drugs in Venezuela and to access free treatment in surrounding countries such as Colombia and Peru, but now these health systems are becoming more cautious due to growing concerns about cost and have recently demanded registration or presentation of passport and visa before treating people. 

## 4. Conclusions—The Future of Migrant Mental Health

This report has hopefully demonstrated that there are clearly a great number of complex challenges to migrant and refugee health. In the face of a changing world and a growing climate crisis, the number of refugees will continue to increase worldwide, no doubt complicated by the scientifically proven effects of climate change on mental health [35,36]. In resource-poor settings, where there is no free care for refugees, currently support is only ever a response to a specific crisis, and therefore there is no long-term solution to ensure migrant health and well-being. This report recommends that a more holistic approach needs to be taken for the prevention of diseases and promotion of migrant health at the global level. Good mental health among refugees should be on the agenda of policy makers and practitioners right from the beginning until the end, because the physical, mental and social dimensions of health cannot be separated. The mental health of refugees is not only important for refugees themselves, but also important for the mental health of host societies, overall social health in the host countries, and the human and financial resources of those host countries. Public leaders and elected officials have a political, social, and legal responsibility to oppose xenophobia and racism that fuels prejudice and exclusion of migrant populations [4]. The positive impacts of migration in advanced economies need highlighting, including how they can enter the workforce to provide medical care, teach children, care for older people, and support understaffed services.

Mental health is not a single achievable objective since there is no quick fix. Therefore, it is essential that there is a continuum of mental health care provisions for all, but particularly for vulnerable groups like migrants and refuges. Given the clear value of cultural mediators in emergency mental health facilities, this group recommends their presence in all facilities and an increase in coverage in those where they already exist. In many settings, migrant and refugee health care considerations and provisions only really cover what is necessary in order to achieve basic human rights in these communities. We must strive to do better, being more ambitious and creative, in order to find innovative mechanisms to enrich the lives of vulnerable populations. Being reactive to urgent situations is not enough. We need to be proactive and inclusive, considering the health of populations and communities as a whole and working together to better them. 

## Figures and Tables

**Table 1:** The five dimensions of access.

Approachability	Acceptability	Availability	Affordability	Appropriateness
Poor access to information on rights, services available and costs of services. There is limited knowledge among irregular migrants about the health system in general	Irregular migrants report limited cultural competence by providers	In some countries, free care is limited to certain facilities, often far from where people live. Difficult to get appointments, hence it is not only the presence but also the capacity of a facility	Several EU countries offer free emergency care, and testing; others offer comprehensive health coverage. However, some offer no cover	This is judged on how well the services provided match the need of the population. Further, adequacy relates to the quality of these services

The five dimensions of access; adapted from [29].

## References

[1] Matlin S.A., Depoux A., Schütte S., Flahault A., Saso L. (2018). Migrants’ and refugees’ health: Towards an agenda of solutions. Public Health Rev..

[2] Bempong N.E., Sheath D., Seybold J., Flahault A., Depoux A., Saso L. (2019). Critical reflections, challenges and solutions for migrant and refugee health: 2nd M8 Alliance Expert Meeting. Public Health Rev..

[3] United Nations High Commissioner for Refugees (UNHCR) Global Trends: Forced Displacement in 2017. https://www.unhcr.org/statistics/unhcrstats/5b27be547/unhcr-global-trends-2017.html.

[4] Abubakar I., Aldridge R.W., Devakumar D., Orcutt M., Burns R., Barreto M.L., Dhavan P., Fouad F.M., Groce N., Guo Y. (2018). The UCL–Lancet Commission on Migration and Health: The health of a world on the move. Lancet.

[5] Aldridge R.W., Nellums L.B., Bartlett S., Barr A.L., Patel P., Burns R., Hargreaves S., Miranda J.J., Tollman S., Friedland J.S. (2018). Global patterns of mortality in international migrants: A systematic review and meta-analysis. Lancet.

[6] Schim S.M., Doorenbos A., Benkert R., Miller J. (2007). Culturally congruent care: Putting the puzzle together. J. Transcult. Nurs..

[7] Schim S.M., Doorenbos A.Z. (2010). A three-dimensional model of cultural congruence: Framework for intervention. J. Soc. Work End Life.

[8] Kelly F., Papadopoulos I. (2009). Enhancing the cultural competence of healthcare professionals through an online course. Divers. Health Care.

[9] Bussema E., Nemec P. (2006). Training to increase cultural competence. Psychiatr. Rehabil. J..

[10] Weech-Maldonado R., Elliott M.N., Pradhan R., Schiller C., Dreachslin J., Hays R.D. (2012). Moving towards culturally competent health systems: Organizational and market factors. Soc. Sci. Med..

[11] Bhugra D. (2004). Migration and mental health. Acta Psychiatr. Scand..

[12] King R. (2002). Towards a new map of European migration. Int. J. Pop. Geog..

[13] Betancourt T.S., Khan K.T. (2008). The mental health of children affected by armed conflict: Protective processes and pathways to resilience. Int. Rev. Psychiatry.

[14] Panter-Brick C., Eggerman M., Gonzalez V., Safdar S. (2009). Violence, suffering, and mental health in Afghanistan: A school-based survey. Lancet.

[15] Hassan G., Ventevogel P., Jefee-Bahloul H., Barkil-Oteo A., Kirmayer L.J. (2016). Mental health and psychosocial wellbeing of Syrians affected by armed conflict. Epidemiol. Psychiatry Sci..

[16] Galper D.I., Trivedi M.H., Barlow C.E., Dunn A.L., Kampert J.B. (2006). Inverse association between physical inactivity and mental health in men and women. Med. Sci. Sports Exerc..

[17] Preibisch K., Hennebry J. (2011). Temporary migration, chronic effects: The health of international migrant workers in Canada. Can. Med. Assoc. J..

[18] Fazel M., Reed R.V., Panter-Brick C., Stein A. (2012). Mental health of displaced and refugee children resettled in high-income countries: Risk and protective factors. Lancet.

[19] Chan E.Y., Mercer S.W., Yue C., Wong S., Griffiths S.M. (2009). Mental health of migrant children: An overview of the literature. Int. J. Ment. Health.

[20] Horlings A., Hein I. (2018). Psychiatric screening and interventions for minor refugees in Europe: An overview of approaches and tools. Eur. J. Psychiatry.

[21] Kaya E., Karadag Caman O., Kilic C., Uner S. (2018). Need for and barriers to accessing mental health care among refugees in Turkey: A mixed methods study. Eur. J. Public Health.

[22] Health of Older Refugees and Migrants. (Technical Guidance on Refugee and Migrant Health). http://www.euro.who.int/__data/assets/pdf_file/0003/386562/elderly-eng.pdf?ua=1.

[23] Nielsen T.R., Vogel A., Riepe M.W., de Mendonça A., Rodriguez G., Nobili F., Gade A., Waldemar G. (2011). Assessment of dementia in ethnic minority patients in Europe: A European Alzheimer’s Disease Consortium survey. Int. Psychogeriatr..

[24] Eurostat: Demography and Migration. https://ec.europa.eu/eurostat/web/population-demography-migration-projections/data/database.

[25] Pancheri C., Roselli V., Todini L., Maraone A., Fioriti V., Mandarelli G., Ferracuti S., Biondi M., Pasquini M., Tarsitani L. (2019). Involuntary psychiatric treatment of first generation immigrants with acute mental disorders in Italy. The role of forced migration. European Psychiatry, 65 Rue Camille Desmoulins, Cs50083, 92442 Issy-Les-Moulineaux.

[26] Tarsitani L., Pasquini M., Maraone A., Zerella M.P., Berardelli I., Giordani R., Polselli G.M., Biondi M. (2013). Acute psychiatric treatment and the use of physical restraint in first-generation immigrants in Italy: A prospective concurrent study. Int. J. Soc. Psychiatry.

[27] Transver. www.transver-berlin.de.

[28] Ambulanzzentrum Kiel. https://www.zip-kiel.de/ZIP_Ambulanzzentrum_Kiel/.

[29] Levesque J.F., Harris M.F., Russell G. (2013). Patient-centred access to health care: Conceptualising access at the interface of health systems and populations. Int. J. Equity Health.

[30] Mladovsky P. (2009). A framework for analysing migrant health policies in Europe. Health Policy.

[31] Orjuela-Grimm M., Fu L., Martinez R., Onesi L., Batista C., Levine J., Barnett M. (2017). Continuity of Oncology Care for Central American Migrant Children in the US. Pediatric Blood & Cancer.

[32] Mijares V.M., Rojas Silva N. Venezuelan Migration Crisis Puts the Region’s Democratic Governability at Risk; Giga Focus Latin America. https://www.giga-hamburg.de/en/publication/venezuelan-migration-crisis-puts-the-regions-democratic-governability-at-risk.

[33] Requena J. (2016). Economy crisis: Venezuela’s brain drain is accelerating. Nature.

[34] Page K.R., Doocy S., Ganteaume F.R., Castro J.S., Spiegel P., Beyrer C. (2019). Venezuela’s public health crisis: A regional emergency. Lancet.

[35] Berry H.L., Bowen K., Kjellstrom T. (2010). Climate change and mental health: A causal pathways framework. Int. J. Public Health.

[36] Page A.L., Howard M.L. (2010). The impact of climate change on mental health (but will mental health be discussed at Copenhagen?). Psychol. Med..

